# Redefinition of Affymetrix probe sets by sequence overlap with cDNA microarray probes reduces cross-platform inconsistencies in cancer-associated gene expression measurements

**DOI:** 10.1186/1471-2105-6-107

**Published:** 2005-04-25

**Authors:** Scott L Carter, Aron C Eklund, Brigham H Mecham, Isaac S Kohane, Zoltan Szallasi

**Affiliations:** 1 Children's Hospital Informatics Program, Harvard Medical School, Boston, MA, 02115 USA; 2 Laboratory of Functional Genomics, Brigham and Women's Hospital, 65 Landsdowne Street, Cambridge, MA 02139, USA

## Abstract

**Background:**

Comparison of data produced on different microarray platforms often shows surprising discordance. It is not clear whether this discrepancy is caused by noisy data or by improper probe matching between platforms. We investigated whether the significant level of inconsistency between results produced by alternative gene expression microarray platforms could be reduced by stringent sequence matching of microarray probes. We mapped the short oligo probes of the Affymetrix platform onto cDNA clones of the Stanford microarray platform. Affymetrix probes were reassigned to redefined probe sets if they mapped to the same cDNA clone sequence, regardless of the original manufacturer-defined grouping. The NCI-60 gene expression profiles produced by Affymetrix HuFL platform were recalculated using these redefined probe sets and compared to previously published cDNA measurements of the same panel of RNA samples.

**Results:**

The redefined probe sets displayed a substantially higher level of cross-platform consistency at the level of gene correlation, cell line correlation and unsupervised hierarchical clustering. The same strategy allowed an almost complete correspondence of breast cancer subtype classification between Affymetrix gene chip and cDNA microarray derived gene expression data, and gave an increased level of similarity between normal lung derived gene expression profiles using the two technologies. In total, two Affymetrix gene-chip platforms were remapped to three cDNA platforms in the various cross-platform analyses, resulting in improved concordance in each case.

**Conclusion:**

We have shown that probes which target overlapping transcript sequence regions on cDNA microarrays and Affymetrix gene-chips exhibit a greater level of concordance than the corresponding Unigene or sequence matched features. This method will be useful for the integrated analysis of gene expression data generated by multiple disparate measurement platforms.

## Background

The first years of microarray analysis of human cancer samples produced several promising results, introducing complex gene expression profiles for diagnostics and predicting disease outcome [1]. However, initial enthusiasm was replaced by uncertainty when classifiers produced for the same type of diseases in various studies shared few if any of the same marker genes [2]. Although microarray results are often reproducible for a single platform, inconsistencies in sensitivity, cross hybridization, and splice variant specificity may render the transfer of results between microarray platforms problematic.

One of the difficulties in the cross platform comparison of microarray data is to ascertain that probes on the various platforms aimed at the same gene do in fact quantify the same mRNA transcript. The various strategies to match probes between different platforms can be constrained by the amount of information provided by the manufacturers of the given microarray. Initially, actual probe sequence information was not released; therefore, probe matching could be based only on gene identifiers such as the Unigene ID. This strategy is known to produce a significant number of incorrect pairings [3]. As partial or complete probe sequence information has become available, more accurate strategies can now be implemented.

In a recent study, we compared several Affymetrix platforms (for which probe sequence information was available) to the Agilent Human 1 cDNA microarray platform [4]. Probe sequence information was unavailable for the Agilent platform except for a 100 base lead sequence at one end of each cDNA probe. Using this information, we queried whether the Affymetrix probes and the 100 base lead sequence could be mapped to a single Unigene transcript. Unigene matched probes across the two platforms that failed this sequence mapping test showed a significantly lower expression correlation across the two microarray platforms [4]. However, the lack of complete cDNA sequence information precluded determination of the actual sequence overlap level with high certainty.

In contrast to the Agilent probes, short sequences from both the 5' and 3' ends are generally available for clones on Stanford cDNA microarrays. Using these sequences to infer the complete clone sequence, we show that the level of probe sequence overlap is highly related to the gene expression concordance between the Affymetrix and cDNA microarray platforms. Eliminating non-overlapping probes allowed us to extract more consistent results from cancer associated gene expression data produced by different platforms and in different institutions.

## Results

Depending on availability or the set of genes to be quantified, large scale gene expression profiling studies have used different versions of chips of a given microarray platform. For the data sets analyzed in this study two types of Affymetrix chips were used: the HuFL oligo chips and the U95Av2 chips. These contain 20 and 16 oligo probes per probe set, respectively. For the cDNA microarray studies, the pool of actual clones shows a very high level of diversity between various studies. Therefore, the exact number of overlapping probes depended on both the specific generation of Affymetrix platform and the set of cDNA clones to which it was mapped. A summary of these data is listed in Table 1.

### Comparison of cDNA and Affymetrix expression measurements

Because cDNA microarray measurements are typically reported as the log ratio of an experimental (Cy5) and control (Cy3) channel, direct comparison with single-channel Affymetrix data required that one of the two data sources be converted to a scale compatible with the other. Because the spot-size on robotically spotted cDNA microarrays can vary substantially, considering only the experimental channel would have given expression measurements prone to probe-quantity artifacts. On the other hand, without direct measurement on the Affymetrix platform of the control RNA used in the cDNA hybridization, it was impossible to replicate exactly the reference response level of each measurement feature.

We attempted to address this difficulty by assuming that the reference RNA batches chosen for each cDNA hybridization uniformly reflect the diversity of experimental transcript populations and therefore that the mean of a gene's measured expression level across all experiments may serve as a reference for the normalization of Affymetrix data (methods). We verified that the mean expression measured by each Affymetrix array did not vary substantially (max- min < 0.25).

### Sequence-overlapping probes give greater cross-platform consistency for the NCI-60 panel

The NCI-60 cell line panel consists of sixty well characterized human tumor cell lines derived from patients with leukaemia, melanoma, and lung, colon, central nervous system, ovarian, renal, breast and prostate cancers. This cell line panel has been developed by the Developmental Therapeutics Program of the National Cancer Institute and routinely used to screen potential anticancer drugs [5].

The gene expression profiles of the NCI-60 cell line panel measured by cDNA microarray and by Affymetrix HuFL oligo chips constitutes a unique data source. To the best of our knowledge, it is the only publicly available dataset in which replicates of a large number of diverse RNA samples have been quantified by these two microarray platforms. Affymetrix microarray probe sets were classified based on their shared sequence identity across the two platforms.

**Figure 1:**
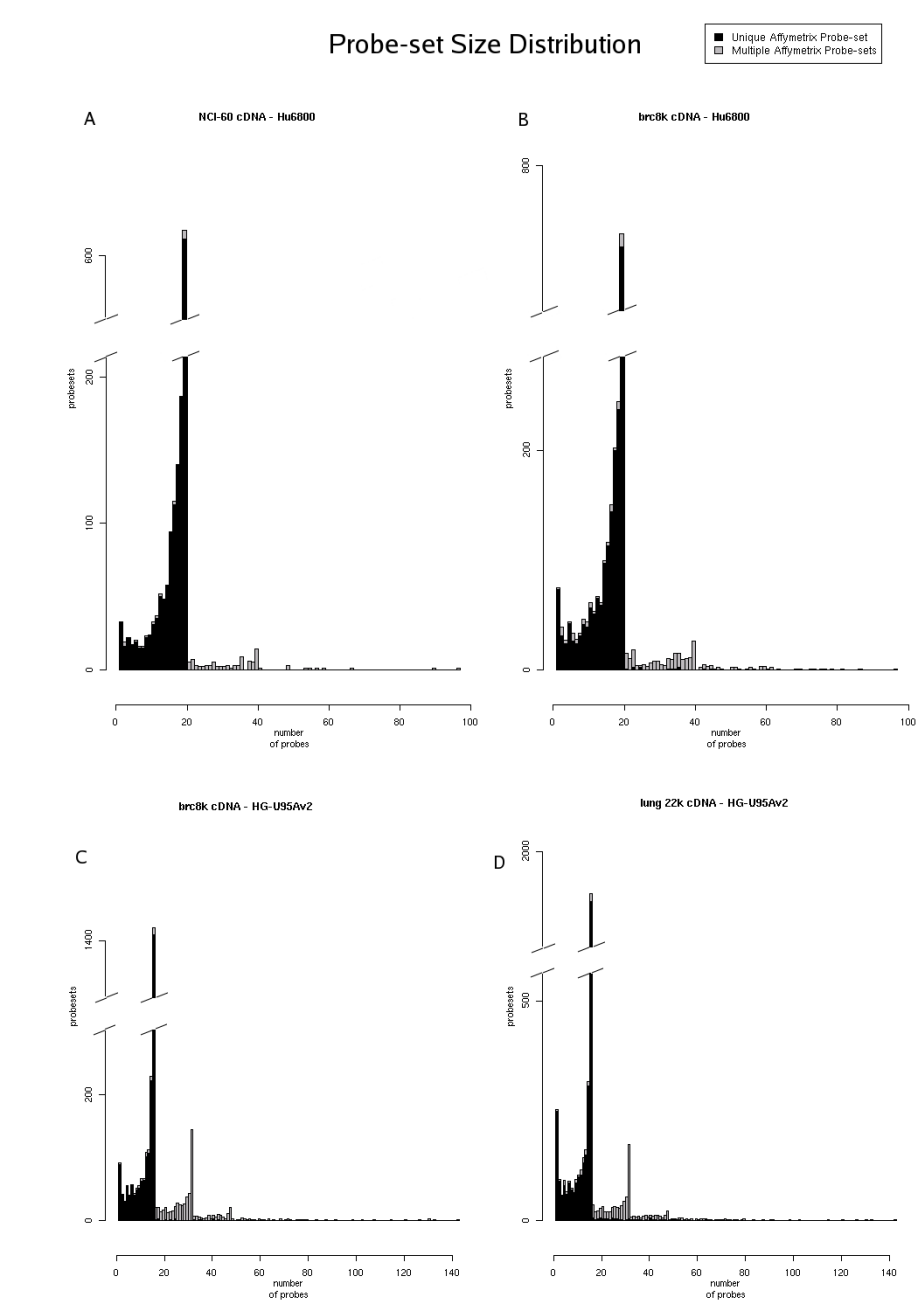
Composition of redefined Affymetrix probe-sets based on overlap with cDNA clone insert sequence. Stacked histograms show the distribution of probe-set size for sets consisting of a single Affymetrix-defined probe-set (black) and for those comprised of probes originally grouped into separate probe-sets by Affymetrix (gray). **A**, NCI-60 10 k cDNA microarray to HuFL alternative CDF. **B**, Breast cancer 8 k cDNA microarray to HuFL alternative CDF. **C**, Breast cancer 8 k cDNA microarray to HG-U95Av2 alternative CDF. **D**, Lung cancer 22 k cDNA microarray to HG-U95Av2 alternative CDF.

Since the actual number of overlapping probes can be between 0 and 20, a large number of potential stratification schemes can be implemented. However, for a clear presentation of results we chose to compare the following classes representing different levels of shared identity: a) Affymetrix probe sets that share a Unigene ID with a cDNA clone. (termed Shared Unigene probes) b) Affymetrix probe sets containing probes that could be sequence-matched to the same transcript sequence as the cDNA clone, but for which no Affymetrix probe actually overlaps the cDNA clone sequence (termed Shared Transcript probes); c) Affymetrix probe sets with 1 to 10 probes sequence overlapping with the cDNA clone (termed Partially Overlapping probes); d) Affymetrix probe sets with 20 (i.e. all) probes sequence overlapping with the cDNA clone (termed Completely Overlapping probes); e) alt-CDF or "redefined probe sets" for which all probes across the entire array that matched to a given cDNA clone insert were used to define a new derivative probe set. This new probe set may contain only a subset (even a single probe) of an original probe set; in other cases probes across several original probe sets were joined into the new derivative probe set (fig 1). For "partially overlapping" and "completely overlapping" probes (classes c and d), the entire original probe set was used for calculating gene expression levels, whereas for the "redefined" probe sets (class e) only the sequence mapped probes were retained.

**Figure 2:**
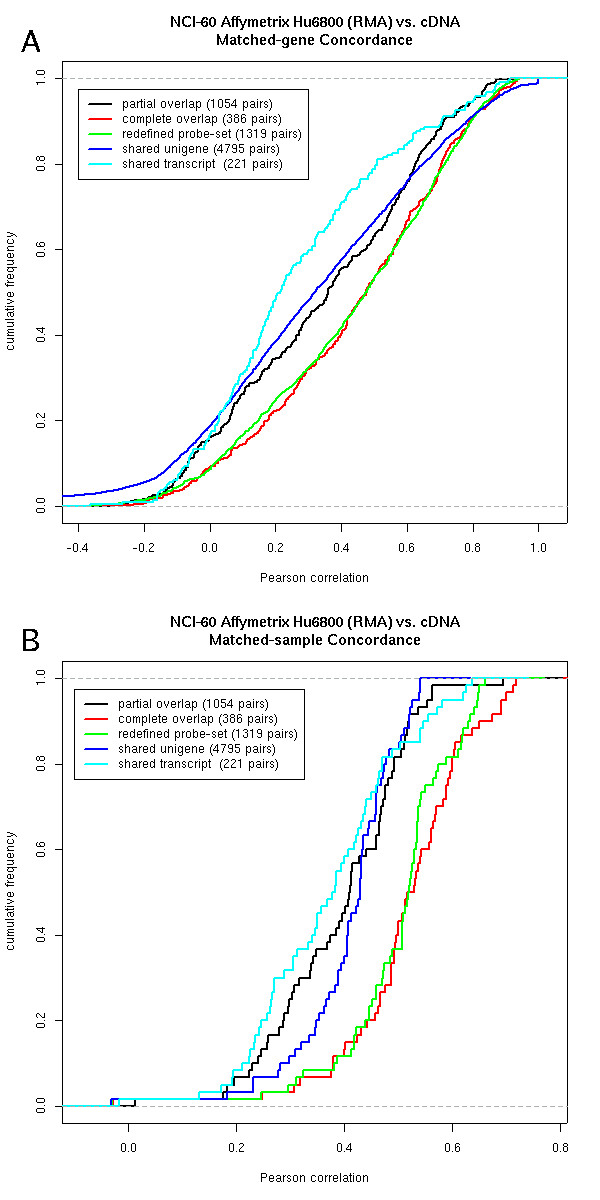
Sequence-overlapping probes give greater cross-platform concordance for the NCI-60 panel. (**A**) Pearson correlation coefficient was calculated for each gene between its expression values measured on the Affymetrix Hu6800 platforms and its expression values measured on the Stanford cDNA microarray across sixty cell lines of the NCI-60 panel. The figure shows the cumulative distribution of the Pearson correlation coefficients for all genes analyzed. The five different curves reflect the level of cross-platform consistency of probe sets with various levels of overlap between the two microarray platforms. Matched gene measurements across the two platforms showed higher correlation when greater numbers of probes in the Affymetrix probe sets overlapped the insert region of the cDNA clone. The highest correlation was attained when only those Affymetrix probes overlapping the insert-sequence of a given cDNA clone were retained. Measurements for which the probes targeted the same transcript as the cDNA clone, but did not overlap the clone sequence, showed the lowest correlation. (**B**), Pearson correlation coefficient was calculated across all genes for each matched sample pair profiled by the Affymetrix Hu6800 platform and by the Stanford cDNA microarray. The figure shows the cumulative distribution of the Pearson correlation coefficients for the sixty cell lines of the NCI-60 panel. Matched cell-line measurements showed identical stratification of correlation levels by feature-matching criteria.

Figure 2 demonstrates the correlation between the Affymetrix and cDNA microarray measurements for the various types of matched probes across the two platforms. Increasing the number of overlapping Affymetrix probes ensures increased cross-platform consistency both for matched genes and matched cell-lines. Additionally, concordance was greatest when only sequence-overlapping probes were used by redefining probe sets, even though in some cases only a single Affymetrix probe was considered. Redefined probes and completely overlapping probes showed the highest concordance levels. (The cumulative correlation distributions showed little difference, however the former method allowed a 4-fold increase in the number of available genes.) These results imply that probes targeting identical transcript sequence regions give substantially stronger concordance than probes that target identical contiguous transcript molecules at different sequence regions. In order to further investigate the effect of direct sequence overlap we examined the performance of Affymetrix probe sets that can be sequence mapped to the same transcript molecule but show no actual overlap with the cDNA clone insert ("shared transcript" probes, class b). These probe sets showed the lowest correlation. This might be due to a number of factors including the presence of splice variants, the probes being subject to different cross-hybridization patterns, or incorrect clone sequence predictions.

**Figure 3:**
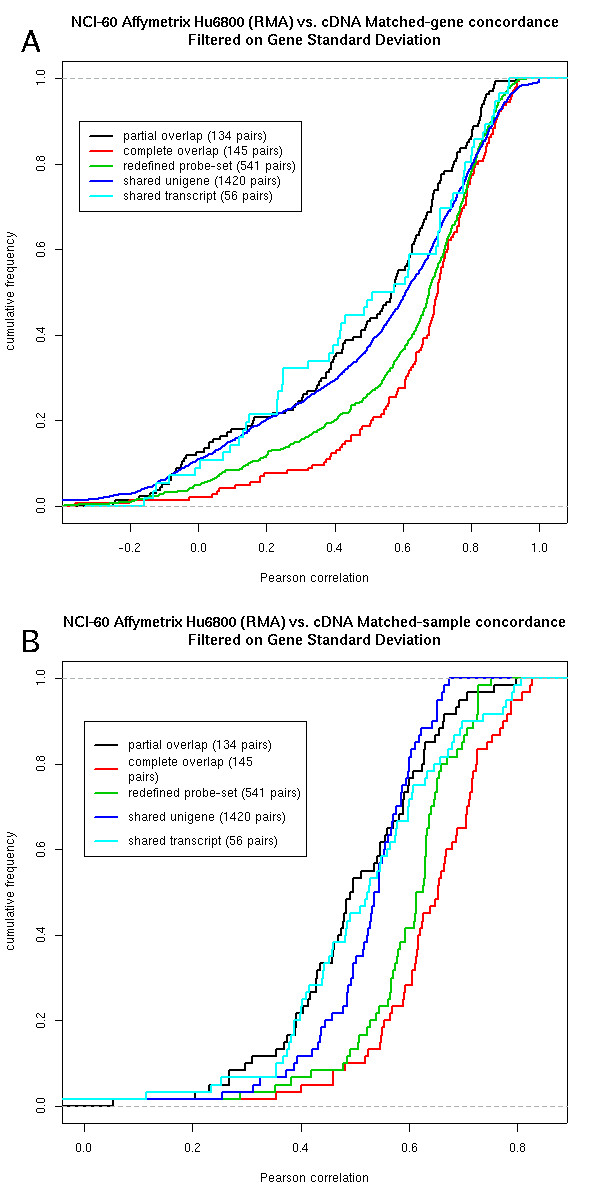
Effect of standard deviation filtering on cross-platform NCI-60 concordance. Genes are filtered removing those with low standard deviations across the 60 cell-lines (methods.) Matching features are determined and concordance assessed as in Figure 1.

Figure 2A also shows, however, that a significant number of probes matched by complete sequence overlap show rather poor correlation (around zero) across the two platforms. The same applies to redefined probe sets. Because we used Pearson correlation as our concordance metric, we expect genes for which the signal fluctuation is below the resolution of the measurement platform to have low levels of concordance, (since the corresponding correlations will be made between noise.) We investigated the effect of removing genes with low levels of variation across the cell-lines on the cross-platform concordance (Fig. 3). Specifically, we removed genes from the Affymetrix dataset with standard deviations below 0.388, (representing the 50^th ^percentile of standard deviation in the full Unigene-mapped dataset.) We removed genes from the cDNA dataset with standard deviations below 0.265, (representing the 50^th ^percentile of standard deviation in the full cDNA dataset.) Matched gene and cell-line concordance was then assessed as described using the genes remaining in both datasets (Fig. 3).

As expected, removing these genes substantially increased both gene and cell-line concordance (Fig. 3). This improvement was substantially greater than that obtained by filtering genes based on mean expression (data not shown). Specifically, the range of median gene correlation increased from approximately 0.2 – 0.4 to 0.4 – 0.6. Interestingly, filtering did not give a substantial improvement near the low end of the distribution, suggesting that some correlations of < 0.1 may be due to incorrect mappings or non-functional probes.

Finally, we noted that "complete overlap" matched pairs performed better than redefined probe sets after standard deviation filtering. This may be due to a number of factors, such as the potentially small number of probes interrogating a given transcript level (in some cases only a single probe.) Alternatively, the redefined probe sets may contain spurious probes in cases where a false-positive clone sequence prediction led to the combination of several Affymetrix-defined probe sets. In any case, the ~4-fold increase in the number of mapped genes available through redefined probe sets may offset the small reduction in concordance.

**Figure 4:**
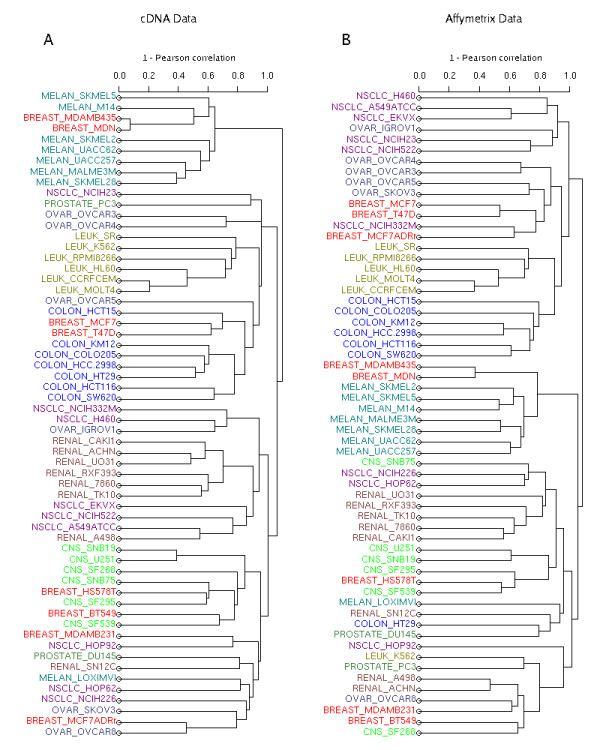
Conserved clustering pattern of the NCI-60 cell lines profiled using cDNA microarray and Affymetrix gene chips. Data was normalized as described (methods). Average linkage Pearson correlation hierarchical clustering was computed for each dataset. Cell line names are colored according to cancer type.

Highly correlated genes are expected to produce a more reproducible unsupervised classification of the cell lines than that derived from a larger pool of genes with less correlation. This can be evaluated in several ways. For example, the hierarchical classification trees derived from the Affymetrix gene chip and cDNA microarray based measurements can be visually compared. Improved reproducibility of classification is indicated by the fact that more cell lines show similar or identical classification on the two hierarchical trees (fig 4).

Encouraged by our initial success, we merged the Affymetrix and cDNA microarray based gene expression profiles and hierarchically clustered the composite data set. More consistent measurements of gene expression across the two platforms would result in a greater number of instances in which the measurements of the same cell-line cluster together. In addition, co-clustering of cell lines of similar origin also provides circumstantial evidence that the gene expression profiles accurately reflect a certain tumor subtype.

**Figure 5:**
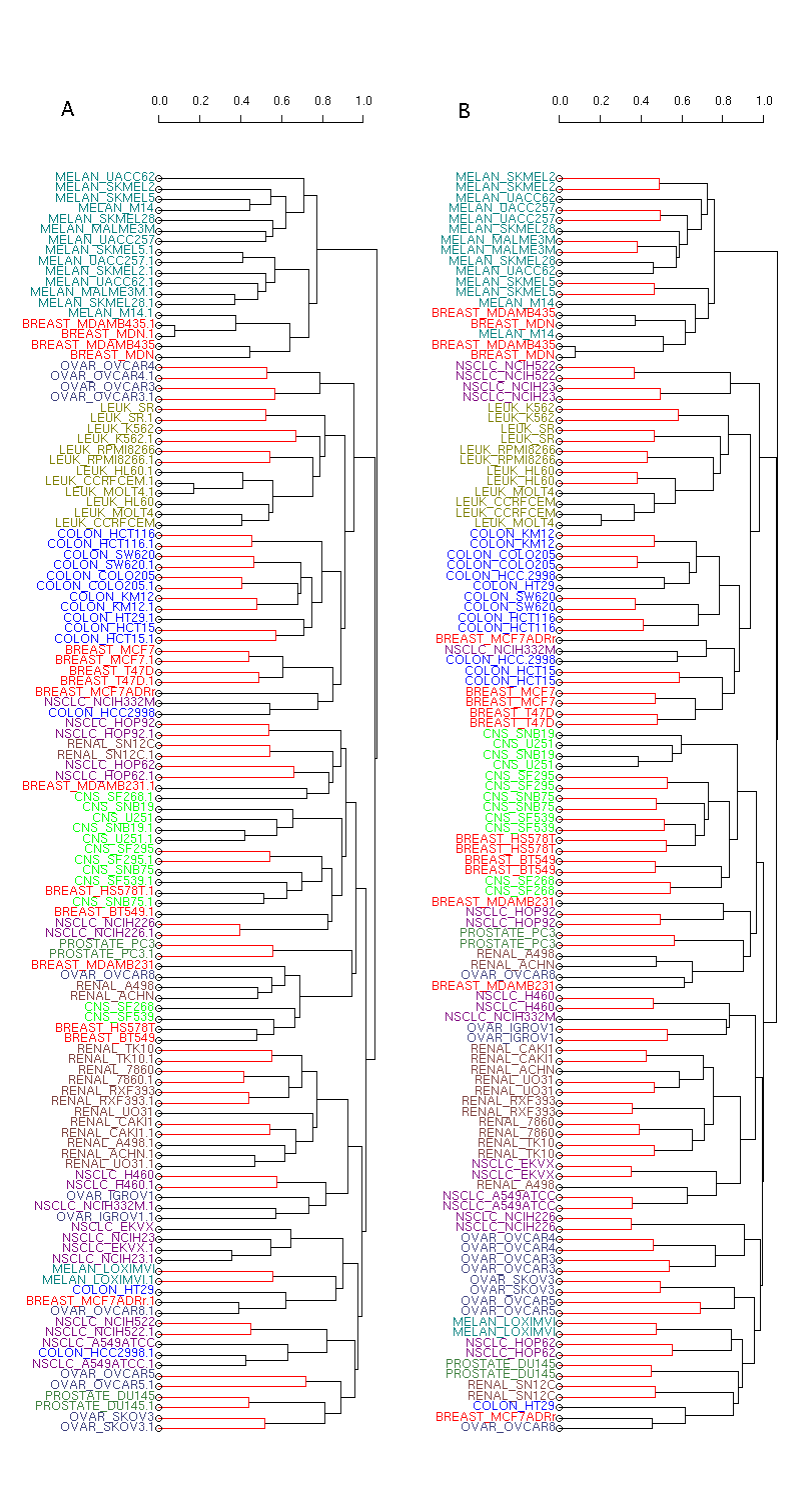
Improved hierarchical clustering of combined NCI-60 cell-lines profiled by Affymetrix gene-chip and cDNA microarray by sequence-overlapping probe measurements. The gene expression profiles obtained for the sixty cell lines by the Affymetrix gene chips and the Stanford cDNA microarray platform were pooled after data transformation as described in the text. Gene expression data by the two different platforms were matched by either Unigene ID matching or by redefining the Affymetrix probe sets based on the sequence overlap criteria of the probes. The pooled gene expression profiles were subjected to average linkage hierarchical clustering. Matched cell-lines from the two platforms which cluster together are marked by red branches in the dendrogram. (**A**) Unigene-matched measurements tended to cluster the cell-lines by measurement platform, and produced only 28 instances of matched cell-lines clustering together. (**B**) Sequence-overlapping probe measurements produced more (43) instances of matched cell-lines from each platform clustering together.

Indeed, hierarchical clustering of the combined datasets resulted in a greater number of matched cell-lines clustering together when only sequence-overlapping measurements were used (fig 5). The majority of matched cell lines are more correlated to one another than to any other cell line from either platform. This was not the case when the expression measurements were Unigene-matched (fig 5A).

We were somewhat disconcerted by the fact that some of the cell lines showed a completely different localization on the two hierarchical trees. For example, the colon cancer cell line HT-29 clusters together with other colon cancer cell lines on the cDNA microarray derived tree but it is placed in a different cluster on the Affymetrix gene chip based classification tree (fig 4). An obvious explanation for this discrepancy would be the failure of the Affymetrix gene chip based measurement. Since no replicates were produced for any of the measurements, there is no statistically sound way of evaluating the quality of any of the gene expression profiles except by some circumstantial measures. For example, most cell lines had cross-platform correlation coefficients larger than 0.2 (Fig 2B). HT-29 was the single outlier with correlation consistently near 0. We obtained an alternative measurement of the same cell line based on an HG-U133A Affymetrix gene chip (a generous gift of Avalon Pharmaceuticals Inc.) We extracted a gene expression profile using the "redefined probe sets" strategy. This gene expression vector produced a much higher correlation coefficient (0.208) with the corresponding cDNA microarray measurements.

### Sequence overlapping measurements improve cross-platform classification of breast cancer subtypes

We were seeking further confirmation for our method using gene expression profiles derived from various human tissue samples. These data sets do not allow highly controlled side-by-side comparisons such as the above presented analysis using * in vitro *cell lines. Therefore, we needed to rely on "indirect" measures of cross-platform consistency, such as classification reproducibility.

Namely, we investigated whether sequence matching of probes would enable us to reproduce the classification of primary breast tumor derived gene expression profiles produced by different microarray platforms.

**Figure 6:**
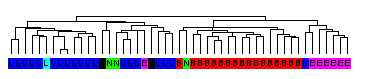
Increased efficiency of breast cancer subtype classification transfer from cDNA microarray to Affymetrix HuFL gene-chip tumor-profiles by sequence-overlapping probe measurements. Tumor samples profiled on the Affymetrix platform were classified according to their correlation with the set of subtype median-centroids derived from cDNA microarray measurements (see methods). The classified samples were then hierarchically clustered using Pearson correlation and average-linkage agglomeration. Affymetrix measurements matched to cDNA centroids by sequence-overlap of probe features produced more coherent classifications than those obtained in the original transfer (Sørlie), specifically, more coherent Luminal A and ERBB2+ subtype clusters.

A breast-cancer subtype classifier was derived from a cohort of patients profiled on cDNA microarrays [1]. This classifier transferred to Affymetrix HuFL gene expression data [6] only to a limited extent [7]. Recently, we improved on those results by using only those Affymetrix and cDNA probes that could be mapped to the same transcript [4]. This earlier publication, however, did not involve the selective use of only those oligo probes that actually matched the cDNA clone. Here we introduced the use of "redefined probe sets" as described in the methods. This was coupled with an advanced normalization method, RMA [8], leading to a strong overall improvement over the original results of Sørlie et al [7] (fig 6). In particular, with two exceptions, all samples could be assigned to a breast cancer subtype defined by the cDNA microarray derived centroids. In addition, more than 70% of all samples clustered in their own well-defined clusters.

**Figure 7:**
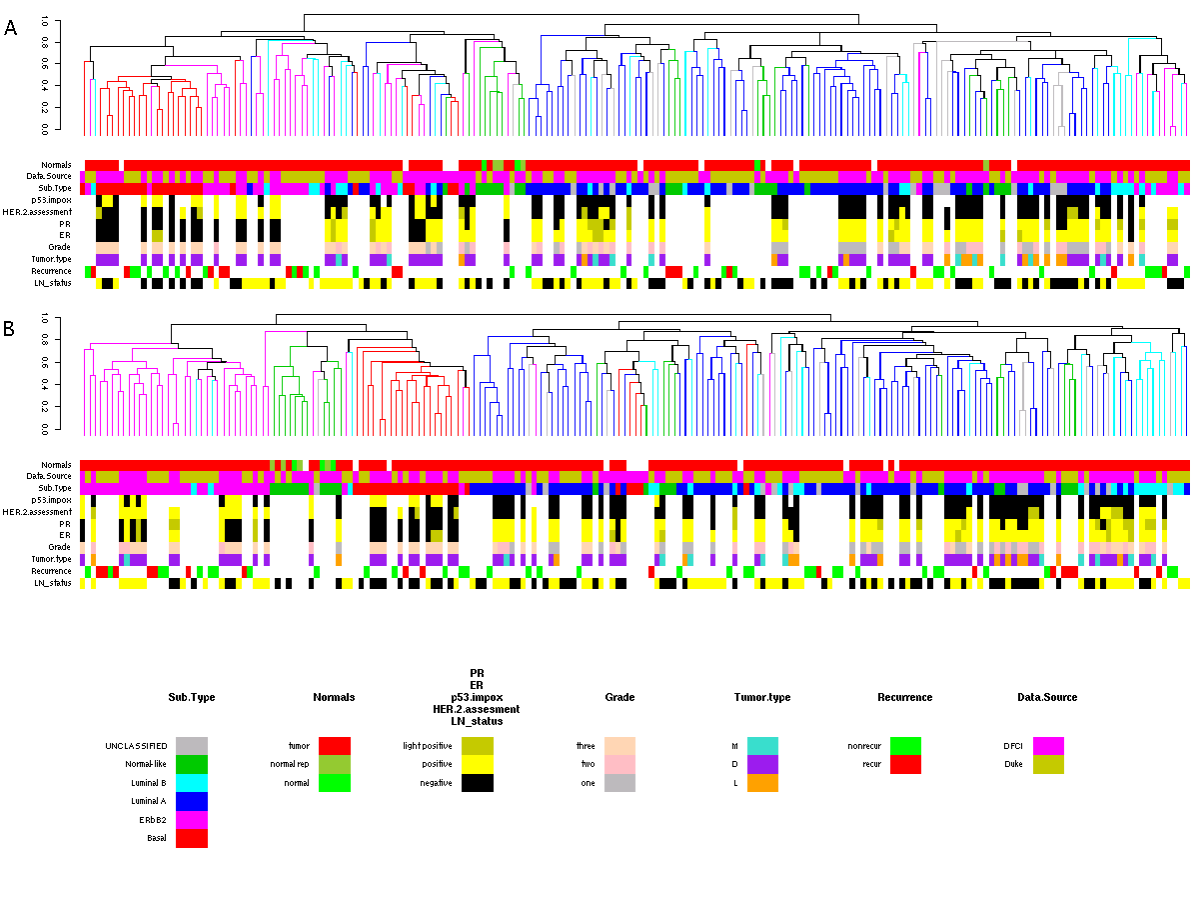
Increased efficiency of breast cancer subtype classification transfer from cDNA microarray to Affymetrix HG-U95Av2 gene-chip tumor-profiles by sequence-overlapping probe measurements. Tumor samples profiled on the Affymetrix platform were classified according to their correlation with the set of subtype median-centroids derived from cDNA microarray measurements (see methods). The classified samples were then hierarchically clustered using Pearson correlation and average-linkage agglomeration. (**A**), Affymetrix measurements matched to the cDNA centroids by Unigene identifier. (**B**), Affymetrix measurements matched to cDNA centroids by sequence-overlap of probe features produced more coherent classifications. In particular, the large ERbB2+ subtype cluster (upper left) is mostly absent from the unigene-based classification. The significance of this cluster is supported by the observation that all tumors in this cluster for which Her-2 amplification was assessed by immunohistochemistry were designated positive.

Furthermore, we compared the transfer of the cDNA-based classifier [7] to two additional cohorts of breast cancer samples profiled on Affymetrix HG-U95Av2 gene-chips [9,10], using both the 'shared Unigene' (fig 7A) and 'redefined probe sets' (fig 7B) to match measurements (see methods). Since true classes are usually not known *a priori* for novel cancer subtypes, we focused our attention on a subtype where gene expression profiles associated with an independent immunohistochemical marker: Her-2 / erbB2 status. Significantly, the classification based on 'redefined probe sets' contains a larger and more coherent ERBB2+ subtype cluster than that based on shared Unigene identifier. The validity of this cluster was substantiated by the immunohistochemical assessment of Her-2 status (available only for the Santorini cohort); all of the tested samples in this cluster stained positive for Her-2 amplification.

### Sequence-overlapping measurements improve cross-platform similarity of normal lung samples

**Figure 8:**
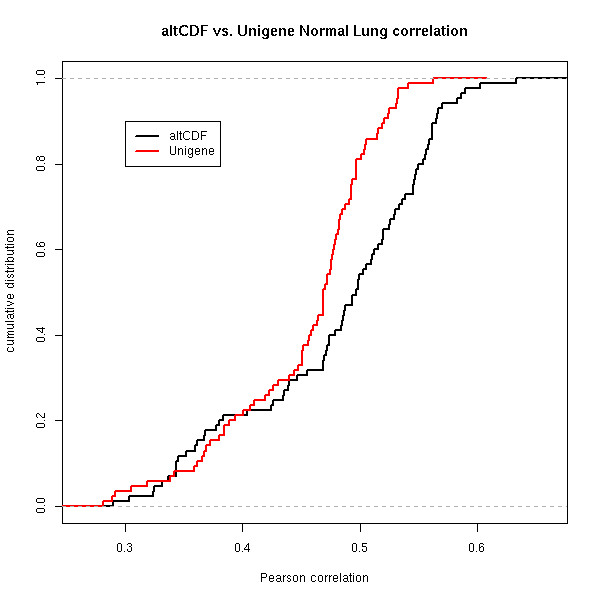
Increased cross-platform similarity of normal lung samples by sequence-overlapping probe measurements. Shown are the cumulative distributions of the 5 × 17 cross-platform sample correlations (see methods.) substantially greater similarity is observed when only sequence-overlapping probe measurements are retained (black curve.)

Finally, we evaluated our sequence-overlap probe set redefinition method on a third cDNA platform. In this case, we evaluated the cross-platform similarity of normal lung samples profiled on cDNA microarrays [11] and Affymetrix HG-U95Av2 gene chips [12]. These two independent data sets contain normal samples from different patients. However, a robust gene expression profile was detected in both studies for the normal lung tissue samples [11,12]. If this robust, normal gene expression profile is accurately measured by both microarray platforms, then a high Pearson correlation coefficient would be expected between the normal samples, independently from the microarray platform used for a given tissue sample. Therefore, we calculated the correlation coefficient between each possible pair of normal gene expression profiles across the two platforms. Two probe matching strategies, the Unigene and sequence-overlap based mappings were compared (fig 8). The significance of the observed increase in cross-platform correlation was assessed at p = 0.0002 (methods), further highlighting the advantage of using only sequence-overlapping measurements for cross-platform comparison.

## Discussion

Despite the fact that all microarray technologies are based on the same basic principle of complementary hybridization, various probe selection strategies aim to achieve optimal probe performance given the technological constraints using fundamentally different strategies. In order to be able to plan long-term microarray based experimental strategies, end users have hoped either for a clearly superior technology to emerge, perhaps supported by a large number of independent validations, or for a high level of cross-platform consistency when the same type of RNA is expression profiled on different platforms. The latter being true would mitigate the risk of committing to a less accurate technology. Unfortunately, this hope has not been fulfilled yet. The limited number of independent validations published so far suggested a similar level of accuracy, or lack thereof, for the most widely used platforms [13-15], and the first cross platform comparison studies revealed an alarming level of inconsistency between platforms such as the cDNA microarray and the Affymetrix oligo chip [16]. This provided little guidance for prospective users on how to choose the technology best suited for their experiments.

Cross platform consistency is an imperfect tool with which to validate microarray platforms. Lack of consistency can be caused by the inferior performance of either one or both platforms, without clear indication of their relative merit. On the other hand, highly similar results across platforms could be simply caused by consistent cross-hybridization patterns without either platform measuring the true level of expression. Nevertheless, a high level of cross platform consistency is desirable. If both platforms perform accurate measurements then cross platform consistency will automatically follow. In other words, cross platform consistency is the *sine qua non *of accurate microarray measurements but by itself will not validate the technology.

Cross platform inconsistencies can be caused by at least two major factors: a) significant differences in noise structure between technologies; b) differential hybridization of homologous probes designed to measure the same gene on various platforms. It has been shown that the most consistent results across different versions of the Affymetrix DNA chips are provided by identical probes [17]. Probes with less or no sequence overlap, even if targeting the same gene at different locations, show substantially lower consistency. Therefore, sequence matching probes provides a strategy for dissecting the sources of cross platform inconsistency.

There are only a few publicly-available data sets that allow comprehensive cross platform comparison of a relatively large number of RNA samples with ample probe sequence information available. The most widely studied of these is the gene expression profiling of the NCI-60 cell line panel produced by the Affymetrix and cDNA microarray technologies [5,16,18-20].

These two data sets showed an alarming level of inconsistencies in an early study when microarray probes, due to the lack of available probe sequence information, were matched across platforms by Unigene IDs [16]. A higher level of consistency was achieved in a subsequent study following the release of probe sequence information by Affymetrix [18]. The authors found a higher level of cross platform consistency using only the subset of probe sets that could effectively be sequence mapped to the same Unigene entity as the corresponding cDNA clone. We obtained similar results in a more limited cross platform comparison study [4]. However, this strategy did not take into consideration whether the short individual oligo probes actually overlapped the corresponding cDNA clone insert. Therefore, portions of the matched Affymetrix probe-sets could have been measuring different regions or different splice variants of the target transcript probed by the cDNA clone. This was perhaps the reason that reproducing the clustering of the NCI-60 cell lines required the highly biased supervised filtering of all genes with a low level of consistency [18]. We introduced here a further improvement that allowed us to rely solely on sequence information and eliminated any further supervised filtering based on expression data. Our strategy relied on using expression signals from only those short individual oligo probes that could be physically mapped onto the corresponding cDNA clone insert. Furthermore, this grouping was done irrespective of the default manufacturer-defined probe sets, in some cases combining probes from several of them. This was much facilitated by a recently introduced elegant computational tool that allows the redefinition of an entire Affymetrix chip definition file within the framework of Bioconductor [21,22]. This strategy constitutes the highest level of sequence based stringency for matching Affymetrix probe sets with cDNA clones to date. Given the importance of correctly designed probes, it is not surprising that this method provides the highest level of cross platform consistency at different levels of the analysis. In addition to the higher levels of correlation, it also improved the transfer of classification results between breast cancer associated gene expression data produced by different microarray platforms.

## Conclusion

We have shown that probes which target overlapping transcript sequence regions on cDNA microarrays and Affymetrix gene-chips exhibit a greater level of concordance than the corresponding Unigene or sequence matched features. Despite these promising results, we should remain aware of the limitations of this method. Microarray signals are a composite of three factors: 1) true signal from the targeted gene, 2) cross-hybridization with other genes, and 3) random noise. The stringent sequence matching applied in this paper increases the consistency of the first two factors across the platforms. However, it does not allow for an easy deconvolution i.e. whether the higher level of observed cross-platform consistency is due to measurement of only the true signal or to reproduction of the cross hybridization pattern. This determination will require further studies underway in our laboratory.

Finally, the assumption that reference mRNA batches used in cDNA hybridizations reflect the full level of diversity in a target experimental mRNA population is imperfect. Without access to measurements of this mRNA on the experimental platform of interest, it is impossible to replicate exactly the normalization inherent in a cDNA log ratio. It is therefore important that the origin of the reference mRNA sample be kept prominently in mind when considering the results of any cDNA microarray experiment.

## Methods

### Inference of cDNA probe sequences

For a given cDNA clone, all corresponding read sequences were extracted from dbEST [23]. When both 5' and 3' read sequences were available for a given clone, these sequences were BLASTed against the Acembly transcript database corresponding to human genome build hg16. The alignment results were used to construct a list of putative insert regions. If both clone read sequences had a high-quality (expectation value < 0.001) hit in the correct sense to a given transcript, the transcript region comprising both read sequences and the flanked region is predicted to be the clone sequence. Statistics for the mapping of each cDNA microarray platform are summarized in table 1.

### Mapping of Affymetrix probes

For a given Affymetrix platform, all probe sequences as obtained from Affymetrix were matched against the Acembly transcript database. Only exact matches were retained. Based on these results, we determined the number of Affymetrix probes in each probe set that overlapped each predicted clone sequence.

In addition to assessing the extent of whole probe set-level overlap with the clone sequence, we also constructed alternative groupings of Affymetrix probes for each platform. These redefined probe sets comprised all Affymetrix probes that overlapped the corresponding cDNA clone, whether or not those probes were intended to be a single probe set by the manufacturer. In some cases, these probes spanned several of the probe sets as defined by Affymetrix (table 1). We then re-computed normalized expression values for the datasets using these redefined probe sets using the "altcdfenvs" package in Bioconductor [21,22]. Applying this strategy allowed us to use only those short oligo probes that overlapped the corresponding cDNA clone insert. The alternate probe mappings are available in a format compatible with the "altcdfenvs" package [see Additional file 1].

### Normalization of Affymetrix data for comparison with cDNA microarray data

All raw Affymetrix probe-level measurements were first transformed into log expression measures using RMA [8]. These expression measurements were then converted into log ratios by subtracting the mean (log) expression from each measurement. In all cases, this process was performed for each sample with respect to its complete original cohort. This was done to minimize artifacts resulting from differences in RNA amplification, labeling, hybridization conditions, etc. cDNA log ratios for each gene were mean centered with respect to the original data set.

### NCI-60 concordance

Normalized cDNA microarray expression data for the NCI-60 cell lines was obtained from a previous study [18]. The reference RNA batch for this study was derived from "12 highly diverse cell lines of the 60" [19]. Raw CEL files were obtained for the same cell lines run on the Affymetrix HuFL oligonucleotide expression platform [20] and normalized as described above.

In addition to sequence-overlap methods of matching measurements across the platforms, we also assessed the weaker criterion of matching probes by Unigene identifiers (build #175). Unigene clusters corresponding to each probe set were obtained from Affymetrix (annotation downloaded September 2004.) Clones on the cDNA microarray were assigned to a Unigene cluster if that cluster included an entry annotated as a read sequence for the clone's IMAGE identifier.

Concordance was assessed by computing the Pearson correlation coefficient between matched-pairs of both genes and cell-lines across the two platforms. Genes were excluded if more than 50 of the cDNA measurements for that gene were missing. We also computed the average-linkage Pearson correlation hierarchical clustering of the combined datasets using both the Unigene and sequence-overlap mappings.

### Breast cancer classification

Previously described cDNA microarray expression measurements from a cohort of breast cancer patients were obtained for an 'intrinsic' gene set used to classify tumor subtypes [7]. The original reference RNA batch used for the cDNA study was derived from 11 different cultured cell lines [24]. The samples were grouped into classes corresponding to the five subtypes, and median centroids were calculated for each class as described [7]. Putative clone sequences for each clone on the microarray were determined as described above.

Raw Affymetrix HG-U95Av2 CEL files were obtained for 199 samples from two additional cohorts of breast cancer patients profiled in previous studies [9,10] and normalized as described above. Each sample was then assigned to the subtype corresponding to the median centroid for which it attained the greatest Pearson correlation level, or was designated "unclassified" if no correlation exceeded 0.1. The quality of the classification produced by both mappings was evaluated by computing the average-linkage Pearson correlation hierarchical clustering of the classified samples, based on the rationale that a more meaningful classification should correspond to more coherent sample-clusters consisting of each subtype.

### Normal lung sample comparison

cDNA microarray data profiling of 5 normal lung samples was obtained from a previous study of lung cancer [11]. The original reference RNA batch used for the cDNA study was derived from 11 different cultured cell lines [24] (the same reference as used in the breast cancer experiment.) Affymetrix HG-U95Av2 CEL files were obtained from an additional lung cancer study [12], 17 of which corresponded to normal lung samples, and normalized as described above. Cross-platform Unigene and sequence-overlap based mappings were constructed as for the previous analyses. Genes were standard deviation filtered as described for the NCI-60 analysis (min cDNA SD = 0.608, min Affy SD = 0.271.) For each mapping, we calculated the Pearson correlation between each of the 5 × 17 cross-platform sample-pairs and compared the cumulative distributions (Fig 8). The significance of the observed improvement in the redefined probe set mapping was quantified using an exact one-sided Kolmogorov-Smirnov test.

## Authors' contributions

SLC participated in conceiving the study, carried out most of the analyses and prepared the manuscript, ACE participated in conceiving the study, carried out parts of the analyses and prepared the manuscript, BHM participated in the experimental design, ISK participated in conceiving the study and preparing the manuscript, ZS originated and conceived the study and prepared the manuscript.

## Supplementary Material

Additional File 1 ZIP of 4 files allowing the remapping Affymetrix probe-sets described in this manuscript. These files can be used with the "altcdfenvs" package in Bioconductor to implement the redefinition of probe-sets based on sequence-matching with each of the 4 cDNA datasets described. Click here for file

## Figures and Tables

**Table 1:** Summary of mapping cDNA microarray features to probes on Affymetrix gene-chips.

	***NCI-60 – HuFL***	***Brc 8k – HuFL***	***Brc8k – U95Av2***	***Lung – U95Av2***
Total cDNA clones	9707	8820	8820	22691
Clones with both reads sequenced	6222	7015	7015	18645
Clones with predicted insert region	4639	6354	6354	14813
Total Probe-sets defined	1765	2403	3103	4597
Total (perfect match) probes on Affymetrix platform	131541	131541	199084	199084
Total mapped probes	26347	37559	48250	70001
Probes mapped to multiple clones	904	3224	4019	26765
Number of probe-sets with > 1 Affymetrix "probe-set" represented	115	310	664	888
